# *Desulfovibrio vulgaris* caused gut inflammation and aggravated DSS-induced colitis in C57BL/6 mice model

**DOI:** 10.1186/s13099-024-00632-w

**Published:** 2024-07-26

**Authors:** Guoxin Huang, Yilin Zheng, Ni Zhang, Guohai Huang, Weijin Zhang, Qingnan Li, Xuecong Ren

**Affiliations:** 1 Clinical Research Center, Shantou Central Hospital, Shantou, China; 2 Metabolic Medicine Center, the Fourth Affiliated Hospital, International Institutes of Medicine, Zhejiang University School of Medicine, Yiwu, China; 3 Department of Blood Purification Center, Shantou Central Hospital, Shantou, China; 4 Department of Rheumatology and Immunology, Shantou Central Hospital, Shantou, China; 5 Department of Geriatrics, Zhejiang Key Laboratory of Traditional Chinese Medicine for the Prevention and Treatment of Senile Chronic Diseases, Affiliated Hangzhou First People’s Hospital, School of Medicine, Westlake University, Hangzhou, China; 6 State Key Laboratory of Quality Research in Chinese Medicine, Macau University of Science and Technology, Macao, China

**Keywords:** Sulfate-reducing bacteria, *Desulfovibrio vulgaris*, Gut microbiota, Short-chain fatty acid, Colitis

## Abstract

**Background:**

Sulfate-reducing bacteria (SRB) is a potential pathogen usually detected in patients with gastrointestinal diseases. Hydrogen sulfide (H2S), a metabolic byproduct of SRB, was considered the main causative agent that disrupted the morphology and function of gut epithelial cells. Associated study also showed that flagellin from *Desulfovibrio vulgaris* (DVF), the representative bacterium of the *Desulfovibrio* genus, could exacerbate colitis due to the interaction of DVF and LRRC19, leading to the secretion of pro-inflammatory cytokines. However, we still have limited understanding about the change of gut microbiota (GM) composition caused by overgrowth of SRB and its exacerbating effects on colitis.

**Results:**

In this study, we transplanted *D. vulgaris* into the mice treated with or without DSS, and set a one-week recovery period to investigate the impact of *D. vulgaris* on the mice model. The outcomes showed that transplanted *D. vulgaris* into the normal mice could cause the gut inflammation, disrupt gut barrier and reduce the level of short-chain fatty acids (SCFAs). Moreover, *D. vulgaris* also significantly augmented DSS-induced colitis by exacerbating the damage of gut barrier and the secretion of inflammatory cytokines, for instance, IL-1β, iNOS, and TNF-α. Furthermore, results also showed that *D. vulgaris* could markedly change GM composition, especially decrease the relative abundance of SCFAs-producing bacteria. Additionally, *D. vulgaris* significantly stimulated the growth of *Akkermansia muciniphila* probably via its metabolic byproduct, H2S, in vivo.

**Conclusions:**

Collectively, this study indicated that transplantation of *D. vulgaris* could cause gut inflammation and aggravate the colitis induced by DSS.

**Supplementary Information:**

The online version contains supplementary material available at 10.1186/s13099-024-00632-w.

## Introduction

Gut microbiota (GM) dysbiosis was proven to be one of the most important pathogenetic factors in gastrointestinal diseases [1–3]. Sulfate-reducing bacteria (SRB), one of the core GM members, possesses the properties of producing hydrogen sulfide (H2S) by dissimilatory sulfate reduction to maintain host health [4]. However, overgrowth of SRB could be usually found in many kinds of gastrointestinal diseases, e.g., inflammatory bowel disease (IBD), irritable bowel syndrome (IBS), celiac disease, and colorectal cancer (CRC) [5–7]. Our previous studies also showed that the overgrowth of SRB could be found in the *Apc*^min/+^ mice model, a widely used model for studying colorectal cancer, and inhibiting the abundance of SRB through herbal medicines could bring benefits for the treatment of CRC [8, 9]. The associated studies suggested that the high levels of H2S, a crucial metabolic product from SRB, is toxic to the gastrointestinal tract and lead to damage to the morphology and function of gut epithelia to accelerate the progression of diseases [10, 11].

*Desulfovibrio vulgaris*, the representative bacterium of the *Desulfovibrio* genus, is recognized as one of the potential pathogenic bacteria based on clinical evidence [12, 13]. Our previous study indicated that the total metabolites of *D. vulgaris* have the capacity to induce mitochondrial dysfunction, thereby impairing the host’s ability to acquire energy [14]. Moreover, a recent study also showed that *D. vulgaris* flagellin (DVF) could exacerbate colitis due to the interaction of DVF and LRRC19, leading to the secretion of pro-inflammatory cytokines [15].

The intricate interplay within GM suggests that the excessive growth of *D. vulgaris* may have a profound impact on the overall health of the host. It is possible that this overgrowth could alter the growth of other bacterial species, disrupting the delicate balance within GM. Additionally, persistent infection with *D. vulgaris* might also cause changes in the morphology and function of gut epithelial cells, further contributing to the development and progression of gastrointestinal diseases. Given the potential implications of *D. vulgaris* overgrowth on host health, it is crucial to conduct more comprehensive studies to evaluate its impact on GM and the development of gastrointestinal diseases.

In this study, we transplanted *D. vulgaris* into mice with DSS (dextran sulfate sodium)-induced colitis and established a one-week recovery period to investigate the impact of *D. vulgaris* during both the progressive and recovery stages. qPCR (quantitative real-time polymerase chain reaction) was carried out to analyze the expression of inflammatory cytokines. Moreover, 16S rRNA amplicon sequencing was used to evaluate the GM composition, and HLPC-Q-TOF/MS (high performance liquid chromatography-quadrupole-time-of-flight mass spectrometry) was performed to test the concentration of short-chain fatty acids (SCFAs).

## Materials and methods

### Animal and treatment

C57BL/6 mice, aged 6–8 weeks, were purchased from the Chinese University of Hong Kong. The animal welfare and experiments procedures were strictly followed the guidelines approved by the Ethics Review Committee of Macau University of Science and Technology. The mice were maintained in an automated, environmentally controlled setting featuring a 12-hour light-dark cycle and provided unlimited access to MilliQ water and PicoLab^®^ Rodent Diet (sourced from LabDiet, USA).

**Fig. 1:**
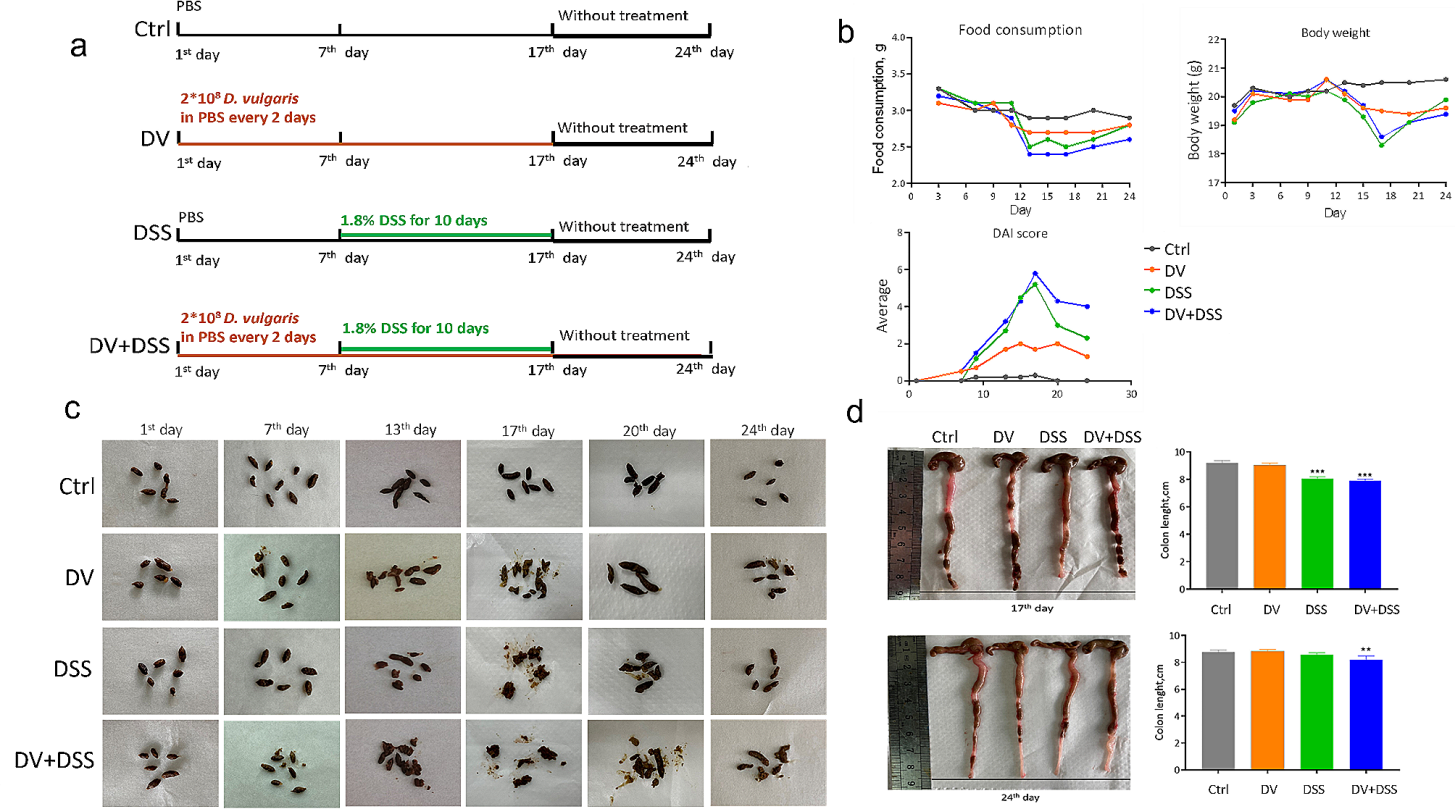
*D. vulgaris* facilitated the manifestation of DSS-induced colitis. (**a**) The treatment schemes. (**b**) The monitoring of food intake, body weight, and Disease Activity Index (DAI) scores throughout the experimental duration. (**c**) The images of fecal consistency during the whole experiment. (**d**) The length of the colon on the day of 17th and 24th. *n* = 8

A total of 32 mice were employed and randomly assigned to four groups: the control (Ctrl) group, the D. vulgaris (DV) group, the Model (DSS) group, and the D. vulgaris + DSS (DSS + DV) group. Prior to modeling, mice in the DV and DSS + DV groups underwent transplantation of 2 × 10⁸ CFU (colony forming units) of *D. vulgaris* suspended in phosphate-buffered saline (PBS) via gavage for a week (from day 1 to 7). Subsequently, mice in the DV, DSS, DSS + DV groups were administered the drinking water with 1.8% DSS (weight/volume) for ten days (from day 8 to 17) to induce colitis. Notably, the transplantation of *D. vulgaris* by gavage continued throughout the modeling period. Finally, a one-week recovery period (from day 18 to 24) was established to investigate the impact of persistent *D. vulgaris* infection (Fig. 1a).

Throughout the entire experiment, parameters including food consumption, body weight, and the disease activity index (DAI, a composite score based on body weight loss, occult blood presence, and stool consistency) were monitored at intervals of two to three days. DAI scores were evaluated following the previous studies [16, 17]. Stool consistency: normal (score = 0), loose (score = 1), diarrhea (score = 2). Blood loss: no loss (score = 0), gross bleeding (score = 1). Appearance: normal (score = 0), hunched (score = 1), starey coat (score = 2), lethargic (score = 3). Body weight (%) loss: none (score = 0), 0–5 (score = 2), 5–10 (score = 2), 10–15 (score = 3), > 15 (score = 4).

In the experiment of evaluating the impact sodium hydrosulfide (NaHS) on GM composition, mice were administered with NaHS (7.5 mg/kg) using intraperitoneal injection for 15 consecutive days. On the 15th day, feces from every single mouse were collected and kept in -80℃ refrigerator for further experiments.

### The culture of bacteria

*D. vulgaris* (DSM 1744) was purchased from China General Microbiological Culture Collection Center. *Akkermansia muciniphila* (BAA-2869) was purchased from American Type Culture Center. Bacteria was cultured in the anaerobic chamber (Whitley A35 Workstation, UK) with 37℃ and 65% humidity. The value of OD 600 nm from micro-spectrometer was used to determine the density of the bacteria.

### Samples collection and DNA extraction

Half of the mice from every group were sacrificed under CO2 anesthetization on the day of 17th and 24th, respectively. And then open the belly to isolate the cecum and colon for further experiment. The isolated colon was dipped into 2 ml cold PBS to collect the colon content. About 2 cm of the colon were separated and kept in the formalin for histology staining. Mucosa from the mice colon and the cecum were collected and kept in the − 80℃ refrigerator for further experiment.

### Histology and immunohistochemical staining

Paraffin sections 5 μm thick were used for hematoxylin and eosin (H&E) staining, Alcian blue staining and immunohistochemical (IHC). Briefly, the section slides were immersed in xylene to remove paraffin, followed by a graded series of ethanol washes to rehydrate the tissue. To enhance antigen accessibility, microwave-mediated heat-induced epitope retrieval was employed. Subsequently, the IHC targets were specifically probed using antibodies against E-Cadherin (diluted 1:200, #3195S, Cell Signaling Technology), occludin (diluted 1:200, #404,700, Invitrogen), and ZO-1 (diluted 1:200, #617,300, Invitrogen). For chromogen development, the LSAB-HRP kit (K0679, DAKO) was utilized, facilitating the visualization of antibody-bound antigens. The section slides were counterstained with a nuclear stain by hematoxylin. After dehydrated, the tissue sections were mounted and viewed under a Leica microscope with a Leica camera (DFC310 FX) and Leica Application Suite software (Version 4.4.0, Switzerland).

### Measurement of SCFAs

Five microliters of 4-Cl-phenylalanine (0.3 mg/mL) was added to the tested samples (50 µl serum), and then 50 µl of the mixture was added into 200 µl of the chilled MeOH. The mixture was centrifuged at 13,000 rpm for 5 min at 4 °C. The supernatant water layer of the mixture was carefully isolated, with the extraction procedure being performed in duplicate. Finally, the supernatant was dried under the nitrogen stream, derived, and then analyzed by HPLC-Q-TOF/MS as previously described [18].

### Total RNA extraction and quantitative reverse transcription polymerase chain reaction (qRT-PCR)

Total RNA was isolated from mucosa using TRNeasy Mini Kit (QIAGEN) following the manufacturer’s instructions. The concentration of RNA was quantified using NanoDrop 2000 C spectrophotometer (Thermo, USA). Then, the first-strand cDNA was synthesized from 2 µg of total RNA using Transcriptor Universal cDNA Master Kit (Roche). qRT-PCR was performed to determine the differentially expressed level of mRNA using Applied Biosystems ViiA™ 7 PCR system (Carlsbad, CA, USA). The specific primer sequences are listed in Table S1. β-actin was used as an internal control. The qRT-PCR was performed using Power SYBR^®^Green PCR Master Mix (Applied Biosystems Inc., Carlsbad, CA, USA) as described [19].

### DNA extraction and 16S rRNA full-length sequencing

Fecal samples from the 17th day were collected and extracted genomic DNA using QIAamp DNA Stool Mini Kit (QIAGEN) following the guidelines by the manufacturer. The purified DNA was kept in the − 80℃ refrigerator, pending 16 S rRNA amplicon sequencing.

The 16S rRNA gene PCR primers with barcode on the forward primer were used in a 35-cycle PCR using the HotStarTaq Plus Master Mix Kit (Qiagen). After amplification, PCR products are checked in 2% agarose gel to determine the success of amplification and the relative intensity of bands. Samples are pooled together in equal proportions based on their molecular weight and DNA concentrations. The PCR pool is then purified using Ampure PB beads (Pacific Biosciences).

The SMRTbell libraries (Pacific Biosciences) are prepared following the manufacturer’s user guide and sequencing performed on the PacBio Sequel following the manufacturer’s guidelines. After completion of initial DNA sequencing, Circular Consensus Sequencing (CCS) was analyzed using PacBio’s CCS algorithm. The CCS algorithm aligns the subreads individually from each template to generate consensus sequences thereby correcting the stochastic errors generated in the initial analysis. In summary, the CCS sequencing data is depleted of barcodes, oriented 5’ to 3’, sequences < 150 bp removed, and sequences with ambiguous base calls removed. Operational taxonomic units (OTUs) were defined by clustering at 3% divergence (97% similarity). Final OTUs were taxonomically classified using BLASTn against a curated database derived from NCBI (www.ncbi.nlm.nih.gov).

### The test of H2S from cecum content

In the anaerobic chamber, 100 mg of cecal content was placed in an autoclaved tube with 300 µL of PBS. After mixture, 100 µL solution was transferred into a well of a 96-well plate, one well per sample. The lid of the 96-well plate was embedded with agarose containing 0.1 M lead acetate. The reaction principle involved the formation of PBS, a brown to black precipitate, through the reaction between S2- and Pb2+. This reaction took place within the 96-well plate in the anaerobic chamber for 2 h at 37℃ and 65% humidity. After the completion of the reaction, the agarose-embedded lid was visualized using the Gel Doc XR + system (Bio-Rad) and subsequently digitized for analysis using Image J 1.54d (National Institute of Health, USA) [20].

### Statistical analysis

One-way ANOVA (for parametric data) and Kruskal–Wallis tests (for non-normal data) were performed to observe significantly among the groups using GraphPad Prism 8.4.3 (GraphPad Software, USA) [21]. The composition of microbiota was analyzed using R platform with vegan package (2.5.7, DOI: 10.32614/CRAN.package.vegan), pheatmap (1.0.12, DOI: 10.32614/CRAN.package.pheatmap), ggplot2 (3.3.2, DOI: 10.32614/CRAN.package.ggplot2), dplyr (1.0.1, DOI: 10.32614/CRAN.package.dplyr), ggpubr (0.4.0, DOI: 10.32614/CRAN.package.ggpubr), scales (1.1.1, DOI: 10.32614/CRAN.package.scales), grid (4.0.2, DOI: 10.32614/CRAN.package.grid), and tidyverse (1.3.1, DOI: 10.32614/CRAN.package.tidyverse). The R packages of vegan, ggplot2, and scales were used to perform alpha diversity analysis. The R packages of pheatmap, scales, and dplyr were used to perform the heatmap analysis. The value of *p* < 0.05(*), *p* < 0.01(**) and *p* < 0.001 (***) denote for all of the statistical analyses.

## Results

### *D. vulgaris* facilitated the manifestation of DSS-induced colitis

To investigate the impacts of *D. vulgaris* on colitis, an experimental scheme was established as described in Fig. 1a. The results showed significant fluctuations in food intake, body weight, and DAI scores in the mice treated with DSS. However, during the recovery phase (from day 17th to 24th ), the mice in the DSS group showed rapid improvement. In contrast, the mice in DV and DV + DSS groups transplanted with *D. vulgaris* still exhibited signs of inflammation (Fig. 1b). The observations from the images of fecal consistency also reflected a similar trend (Fig. 1c). Moreover, a shorter colon length could be found in DSS and DV + DSS groups when compared to the control group at 17th day, but only in DV + DSS group at 24th day (Fig. 1d).

### *D. vulgaris* worsen the damage of gut barrier induced by DSS

**Fig. 2:**
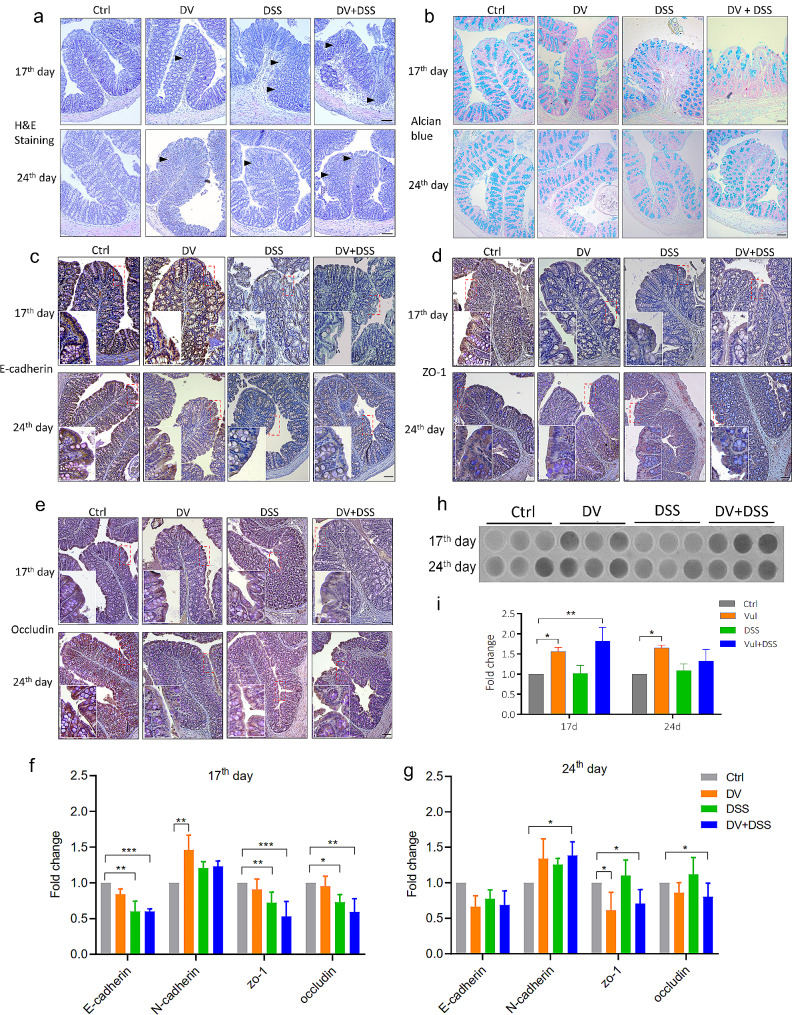
*D. vulgaris* worsen the damage of gut barrier induced by DSS. (**a**) H&E staining on the 17th and 24th days. (**b**) Alcian blue staining on the 17th and 24th days. (**c-e**) IHC results of E-cadherin, occluding, and ZO-1 on the 17th and 24th days. (f&g) mRNA expression of E-cadherin, N-cadherin occluding, and ZO-1 on the 17th and 24th days by qPCR. (h&i) The measure of H2S in cecum content. Scale bar: 100 μm. *n* = 3. *, *p* < 0.05; **, *p* < 0.01

H&E and alcian blue staining were used to evaluate the gut morphology and mucus secretion of the mice exposed to DSS and *D. vulgaris*. The findings revealed that DSS significantly disrupted gut epithelial morphology and reduced mucus secretion on the 17th day, with a subsequent rapid recovery observed by the 24th day (Fig. 2a&b). Conversely, the combination of DV and DSS led to persistent epithelial damage and reduced mucus levels on both the 17th and 24th days. Notably, mice in the DV group exhibited damaged gut morphology and decreased mucus secretion (Fig. 2a&b). To evaluate the gut epithelial barrier integrity, E-cadherin, N-cadherin, Occludin, and ZO-1 were analyzed. Immunohistochemical (IHC) analysis indicated that *D. vulgaris* exacerbated DSS-induced damage to the gut epithelial barrier (Fig. 2c-e). Moreover, qPCR quantification of the mRNA expression of these markers showed that *D. vulgaris* decreased the expression of E-cadherin, Occludin, and ZO-1 while enhancing N-cadherin expression on both the 17th and 24th days. Notably, *D. vulgaris* further aggravated the DSS-induced downregulation of E-cadherin, Occludin, and ZO-1 (Fig. 2f&g). Additionally, we also measured the synthesis of H2S in the cecum, and the results showed the higher level of H2S in DV and DV + DSS groups when compared to the control group on both the 17th and 24th days (Fig. 2h&i).

### *D. vulgaris* exacerbated the gut inflammation induced by DSS

**Fig. 3:**
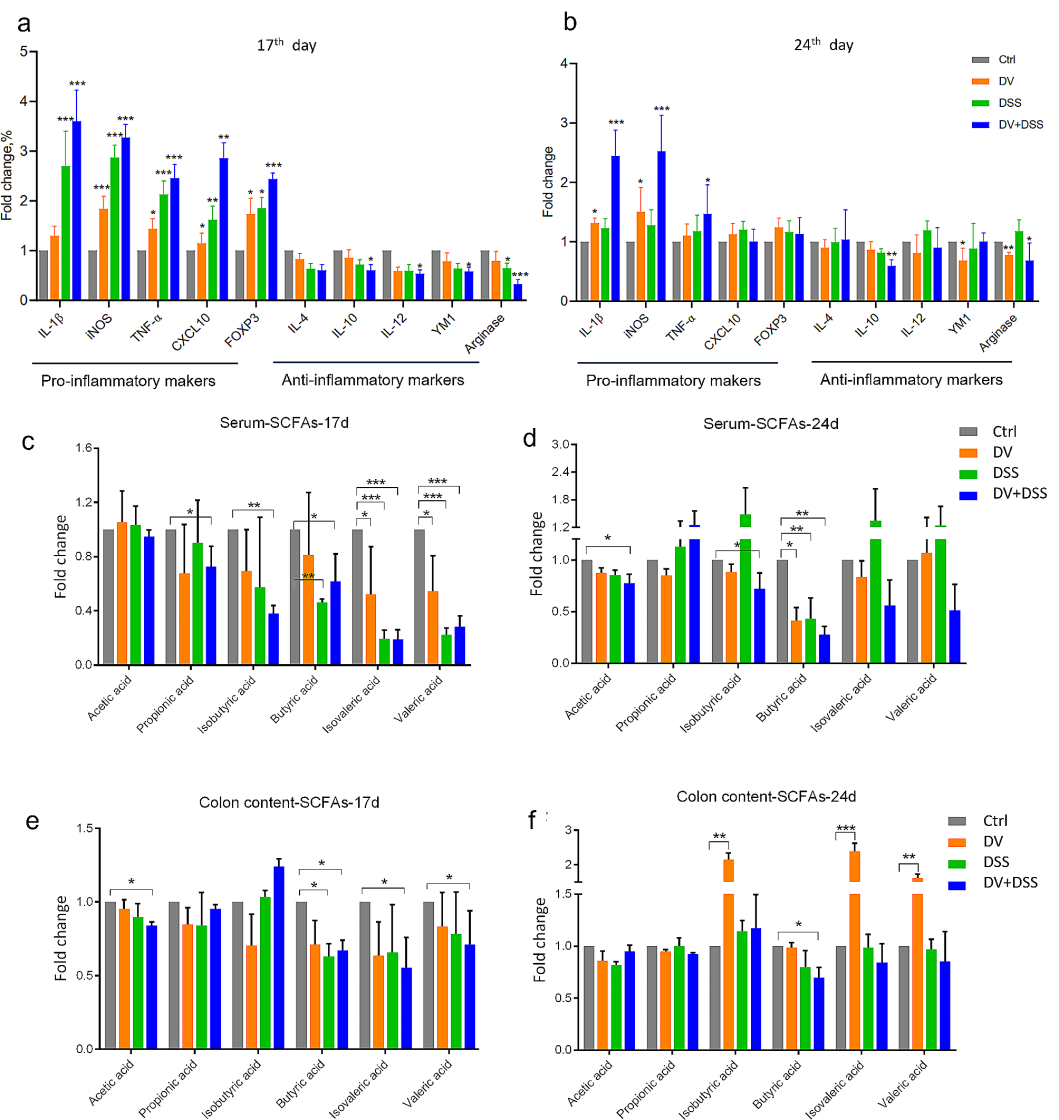
*D. vulgaris* exacerbated the gut inflammation induced by DSS. (**a**) The measure of the expression of inflammatory cytokines on the 17th day by qPCR. (**b**) The measure of the expression of inflammatory cytokines on the 24th day by qPCR. (**c**&**d**) The measure of the serum SCFAs on the 17th and 24th day by HPLC-Q-TOF/MS. (**e**&**f**) The measure of the colon content SCFAs on the 17th and 24th day by HPLC-Q-TOF/MS. *n* = 3. *, *p* < 0.05; **, *p* < 0.01; ***, *p* < 0.001

DSS could significantly imbalance the gut microenvironment by impacting the secretion of inflammatory cytokines. Here, qRT-PCR was used to measure the expression of pro-inflammatory and anti-inflammatory cytokines. The results showed that *D. vulgaris* could potentiate the secretion of pro-inflammatory markers, for instance, IL-1β, iNOS, TNF-α, and FOXP3, but decrease the secretion of anti-inflammatory markers, including IL-4, IL-10, YM-1 (also known as chitinase-like protein 3), and arginase 1 on the 17th day, which induced by treatment of DSS (Fig. 3a). However, by the 24th day, mice in DSS group showed similar secretion of inflammatory markers with the control group. Conversely, the mice in DV and DV + DSS groups exhibited higher expression of pro-inflammatory markers, e.g., IL-1β, iNOS, and TNF-α, but lower anti-inflammatory markers, e.g., IL-10 and arginase 1 when compared to the other two groups (Fig. 3b). These observations suggest that *D. vulgaris* may exacerbate DSS-induced inflammatory responses, leading to a further imbalance in the gut microenvironment.

SCFAs were proven to possess many bioactivities, especially the property of anti-inflammation. Here, HPLC-Q-TOF/MS results showed that *D. vulgaris* could aggravate the decreased concentration of SCFAs from serum and colon content induced by DSS on the 17th day, e.g., butyric acid, isobutyric acid, valeric acid, and isovaleric acid (Fig. 3c&e). Moreover, on the 24th day, the level of certain serum SCFAs in the DSS group recovered to similarity with the control group, for instance, isobutyric acid, valeric acid, and isovaleric acid, but still retained the lower level in the DV + DSS group when compared to control group (Fig. 3d). Additionally, the results also presented the remarkable increase of isobutyric acid, valeric acid, and isovaleric acid in colon content in the DV group on the 24th day (Fig. 3f).

### *D. vulgaris* changed the core GM composition and its associated metabolic pathways of the mice

**Fig. 4:**
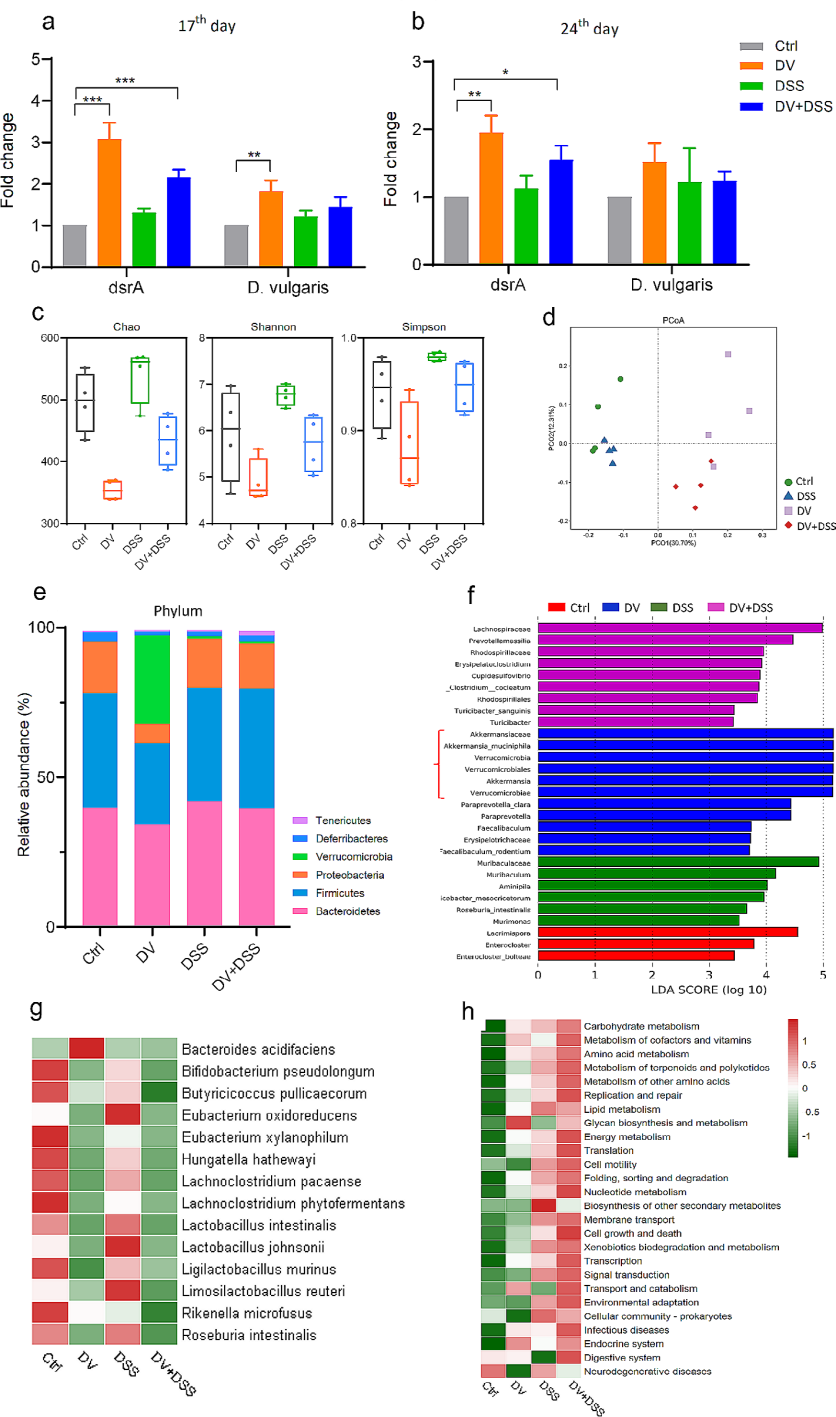
*D. vulgaris* changed the core GM composition and its associated metabolic pathways. (**a**&**b**) The abundance of SRB and *D. vulgaris* on the 17th and 24th days using qPCR. (**C**) The analysis of alpha diversity. (**d**) PCoA for the different groups. (**e**) Average relative abundance of the dominant phyla. (**f**) LDA score among the groups. (**g**) Heatmap analysis for the bacteria associated with SCFAs-producing and anti-inflammation among the groups. The relative abundance (≥ 0.05) of each specie was included for heatmap analysis. (**h**) Heatmap analysis for the microbial KEGG enrichment pathways among the groups. The counts of each enriched KEGG pathway were included for heatmap analysis. For Fig. 4a&b, *n* = 3. For Fig. 4c-h, *n* = 4. *, *p* < 0.05; **, *p* < 0.01; ***, *p* < 0.001

qPCR was used to assess the abundance of SRB and *D. vulgaris* on the 17th and 24th days following transplantation. The expression of the bacterial dissimilatory sulfate reductase (*dsrA*) gene that is crucial for the production of H2S was used to confirm the abundance of the SRB. We found that mice in DV and DV + DSS groups displayed significantly higher abundance of SRB and *D. vulgaris* when compared to the control group on the 17th day (Fig. 4a). Moreover, by the 24th day, mice in DV and DV + DSS groups continued to exhibit high abundance of SRB, and mice in DV group maintained the high abundance of *D. vulgaris* (Fig. 4b). These results indicated that the transplanted *D. vulgaris* could successfully colonize in the gut of the treated mice.

To investigate the change of GM caused by *D. vulgaris* and DSS, 16S rRNA full-length sequencing was performed to analyze GM composition among the groups on the 17th day. Alpha diversity, including Chao 1, Shannon, and Simpson indexes, showed that *D. vulgaris* and DSS could markedly change the GM richness and evenness (Fig. 4c). Moreover, the result of PCoA (principal co-ordinates analysis) also revealed that mice in DV and DV + DSS groups exhibited a more similar GM composition, and distinct from the control and DSS groups (Fig. 4d). Furthermore, phyla comparison showed the different patterns of GM composition among the groups, and the phylum of Verrucomicrobia was remarkably accumulated in DV group when compared to the Ctrl, DSS, and DV + DSS groups (Fig. 4e). Further analysis using LDA (linear discriminant analysis) scores confirmed that *Akkermansia muciniphila*, a member of Verrucomicrobia, was the domain bacterium for the accumulated Verrucomicrobia in DV group (Fig. 4f).

Considering the downregulation of SCFAs concentration and the pro-inflammatory cytokines expression in the treated groups, bacteria associated with SCFAs-producing and anti-inflammation were selected for further analysis, and the results showed that the relative abundance of the bacteria, for instance, *Bifidobacterium pseudolongum*, *Butyricicoccus pullicaecorum*, *Eubacterium xylanophilum*, *Lactobacillus johsonnii*, and *Roseburia intestinalis* were markedly decrease in DV, DSS, and especially in DV + DSS groups (Fig. 4g). Moreover, KEGG (Kyoto Encyclopedia of Genes and Genomes) pathway enrichment analysis results showed that a series of microbial pathways, for instance, carbohydrate metabolism, amino acid metabolism, lipid metabolism, and infectious diseases pathways could be remarkedly accumulated in DSS and DV + DSS groups (Fig. 4h).

### H2S, a metabolite of *D. vulgaris*, stimulated the growth of *A. muciniphila* in vivo

**Fig. 5:**
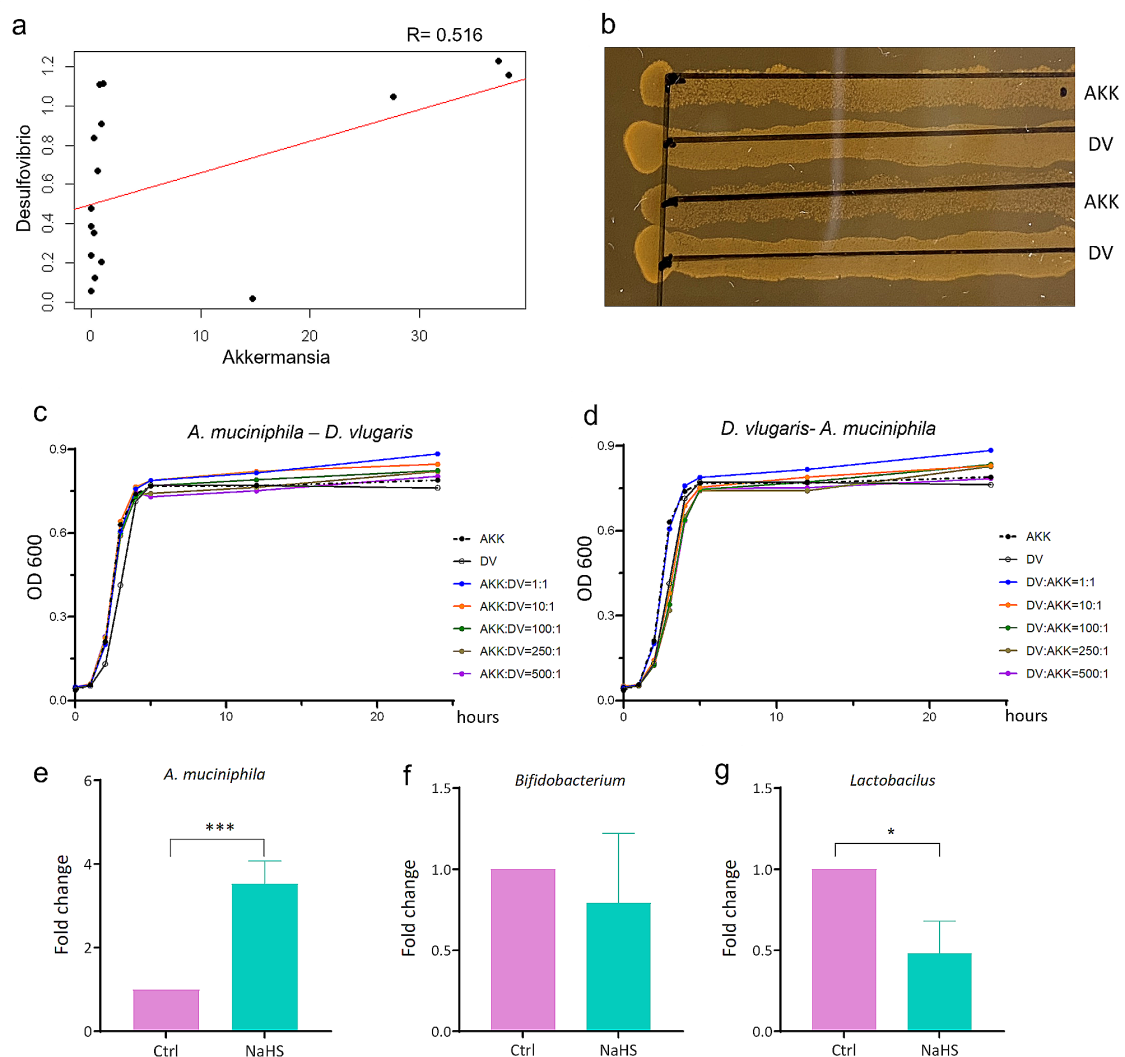
The relationship between *A. muciniphila* and *D. vulgaris*. (**a**) Pearson’s correlation analysis for the relative abundance of genera of *Desulfovibrio* and *Akkermansia* from 16 S full-length sequencing. (**b**) Co-culture of *A. muciniphila* and *D. vulgaris in vitro* in a BHI-agar plate. (**c**) The time course of co-culture of *A. muciniphila* and *D. vulgaris in vitro*, the ratio of *A. muciniphila/D. vulgaris* was from 1:1 to 500:1. (**d**) The time course of co-culture of *D. vulgaris* and *A. muciniphila in vitro*, the ratio of *D. vulgaris/ A. muciniphila* was from 1:1 to 500:1. DV, *D. vulgaris.* AKK, *A. muciniphila.* (**e-g**) Assess the different abundance of bacteria in the mice treated and untreated NaHS using qPCR. *n* = 3. *p* < 0.05; **, *p* < 0.01; ***, *p* < 0.001

Based on the results that treated with *D. vulgaris* could significantly increase the relative abundance of *A. muciniphila* (Fig. 4e&f), Pearson’s correlation analysis was used to determine the relationship between the genera of *Desulfovibrio* and *Akkermansia* from 16S rRNA full-length sequencing. The results exhibited a positive correlation between these two bacteria (*R* = 0.516) (Fig. 5a). To further discuss the potential relationship between *D. vulgaris* and *A. muciniphila*, we co-cultured these two bacteria in the BHI (Brain and Heart infusion)-agar medium. Unfortunately, the outcomes could not conclusively reflect the mutualistic relationship between these bacteria, but still indicated that they could co-exist in the same culture system without competitive interactions (Fig. 5b). Moreover, to gain insight into the relationship between these bacteria, co-culturing in BHI broth was also performed. Notably, the group maintained at a 1:1 ratio of *D. vulgaris* to *A. muciniphila* exhibited a higher cell count compared to the other groups, indicating a possible synergistic effect when these bacteria are present in equal proportions (Fig. 5c&d). Additionally, we also used sodium hydrosulfide (NaHS), which could be metabolized to H2S in vivo, to treat the mice by intraperitoneal injection. Consistent with our previous findings (Fig. 4E&F), the treated mice exhibited an increased relative abundance of *A. muciniphila*, but lower *Bifidobacterium* and *Lactobacillus* when compared to the untreated mice (Fig. 5e-g).

## Discussion

Accumulating evidence shows that SRB closely relates to the development and progression of various diseases, especially the gastrointestinal system, for instance, IBD, IBS, and CRC. SRB, considered as a potential pathogen when they overgrowth in gastrointestinal tract, could be found in many pre- and clinical studies [6, 7, 10, 12]. Most of the previous studies suggested that high level of H2S from the overgrowth of SRB could accelerate the progression of diseases by its toxicity to damage the morphology and functions of epithelial barrier. The potential mechanisms underlying the exacerbation of gut inflammation by H2S involve its ability to modulate gut pH levels and activate the NF-κB signaling pathway [11]. Additionally, studies have highlighted the notable inflammatory effects triggered by LPS (lipopolysaccharide) derived from *Desulfovibrio desulfuricans* [22, 23]. Furthermore, a report showed the pivotal role played by the outer membrane vesicles secreted by *Desulfovibrio fairfieldensis* in contributing to inflammation and disrupting tight junction barrier function [24]. In this study, we found that transplantation of *D. vulgaris* alone could cause gut inflammation, and *D. vulgaris* also significantly aggravated the manifestations of colitis induced by DSS. Moreover, after a one-week recovery period, the mice transplanted with *D. vulgaris* still displayed gut inflammation and epithelial morphology disruption when compared to the control and DSS groups. Mechanistically, treated with *D. vulgaris* could decrease the expression of mucus, E-cadherin, as well as tight junctions, e.g., ZO-1 and occluding, to destroy gut epithelial barrier. Furthermore, *D. vulgaris* caused the downregulation of SCFAs both on 17th day and 24th day, especially butyric acid, isobutyric acid, and valeric acid. Additionally, we also found that transplanted with *D. vulgaris* could markedly change GM composition, for instance, SCFAs-producing bacteria, e.g., *B. pseudolongum*, *B. pullicaecorum*, *E. xylanophilum*, *L. johsonnii*, and *R. intestinalis* were significantly decreased in DV and DV + DSS groups. SCFAs is an important kind of bacterial product which possesses many bioactivities, especially anti-inflammatory, anti-cancer, and immunomodulatory properties, displaying health-promoting effects [25, 26]. Reports also showed that intestinal microbiota-derived SCFAs could regulate immune cell IL-22 production to maintain gut homeostasis to reduce the gut inflammation [27]. Although reports showed that *D. vulgaris* could produce acetic acid via the pyruvate-acetyl coenzyme A pathway [28], we could not find the elevation of acetic acid in this study that might be attributed to inhibited growth of most of the SCFAs-producing bacteria when the overgrowth of *D. vulgaris.* In this study, our findings about the decrease of SCFAs in DV and DV + DSS groups probably was the main mechanism that transplantation of *D. vulgaris* caused the gut inflammation and exacerbated the colitis induced by DSS.

Considering the colonization of SRB might cause persistent inflammation after we transplanted *D. vulgaris* into the mice, we established a one-week recovery period to investigate the impact of SRB on the gut microenvironment. The results showed that mice in DSS group exhibited a restoration to normalcy in terms of gut barrier function, inflammatory cytokine secretion, and SCFAs levels, which are similar to the control mice. However, mice in DV and DV + DSS groups still displayed the disrupted gut barrier, elevated pro-inflammatory cytokines, reduced anti-inflammatory cytokines, and decreased SCFAs level when compared to mice in control and DSS groups. These results implied that the consistent infection of SRB probably led to gut inflammation and aggravated the DSS-induced colitis.

In this study, we also investigated whether *D. vulgaris* transplantation might affect the growth of other bacteria. And the results showed that the growth of *A. muciniphila* is profoundly influenced by *D. vulgaris* colonization (Fig. 4e&f). We next found a positive correlation between *A. muciniphila* and *D. vulgaris* using the 16S rRNA full-length sequencing data. Although our in vitro studies did not yield conclusive evidence of a mutualistic relationship between these bacteria, we observed that they could coexist in the same culture system. Notably, a culture with a 1:1 ratio of *D. vulgaris* to *A. muciniphila* exhibited a higher cell count compared to single cultures of these bacteria. To further investigate the potential mechanism underlying this interaction, we treated mice with NaHS, a compound that metabolizes to H2S in vivo, for 15 consecutive days. The results showed that NaHS could significantly stimulate the growth of *A. muciniphila*. These findings indicated that *D. vulgaris* could stimulate the growth of *A. muciniphila* via H2S in vivo. It is well known that *A. muciniphila* now is considered a next-generation of probiotic [29], and many related references showed its benefits for host health [30, 31]. But several research showed that high relative abundance of *A. muciniphila* also has been found in different diseases, suggesting the complex roles of *A. muciniphila* in progression of diseases [32, 33]. Researchers in this field emphasize the need for more studies to elucidate the roles of *A. muciniphila* in diverse diseases and host backgrounds. Our study might provide valuable insights into the intricate interactions between gut bacteria in this area. However, we also acknowledged that this study just disclosed the positive relationship between the growth of *D. vulgaris* and *A. muciniphila*, the underlying mechanisms remain to be elucidated through further research.

## Conclusion

Collectively, the findings of this study showed that transplantation of *D. vulgaris* could cause gut inflammation and aggravate the colitis induced by DSS. *D. vulgaris* could damage the gut epithelial barrier, increase the secretion of pro-inflammatory cytokines and decrease the secretion of anti-inflammatory cytokines. *D. vulgaris* also hindered the intestinal recovery of the mice post-acute inflammation. Transplantation of *D. vulgaris* changed GM composition, notably reducing the relative abundance of SCFAs-producing bacteria, which led to a decrease in SCFAs level. Moreover, our study firstly found that *D. vulgaris* and *A. muciniphila* could be co-cultured in the same system in vitro, and *D. vulgaris* stimulated the growth of *A. muciniphila* through its metabolic byproduct-H2S *in vivo.* This study revealed the important role of SRB during the process of IBD. Inhibiting the over-growth of SRB might be a potential strategy for prevention and therapy of IBD.

### Electronic supplementary material

Below is the link to the electronic supplementary material.

Supplementary Material 1

## Data Availability

The authors declare that all data supporting the findings of this study are available within the paper, and any raw data can be obtained from the corresponding author upon request.

## Abbreviations

GM: Gut microbiota
H2S: Hydrogen sulfide
SRB: Sulfate-reducing bacteria
IBD: Inflammatory bowel disease
IBS: Irritable bowel syndrome
CRC: Colorectal cancer
DSS: Dextran sulfate sodium
DVF: D. vulgaris flagellin
SCFAs: Short-chain fatty acids
NaHS: Sodium hydrosulfide
CCS: Circular consensus sequencing
DAI: Diseases active index
PCoA: Primary co-ordinates analysis
LDA: Linear discriminant analysis
