# Supplementary material for: Desulfovibrio vulgaris caused gut inflammation and aggravated DSS-induced colitis in C57BL/6 mice model
Source: Gut Pathog. 2024 Jul 26;16:39. doi: 10.1186/s13099-024-00632-w (PMC11282857; doi:10.1186/s13099-024-00632-w)
Supplement: Supplementary file 1 — Supplementary Material 1 [file 13099_2024_632_MOESM1_ESM.docx]

***Desulfovibrio vulgaris* caused gut inflammation and** ***Desulfovibrio vulgaris* caused gut inflammation and aggravated DSS-induced colitis in C57BL/6 mice model**

Guoxin Huang^1,6,#,*^ Yilin Zheng^1,#^, Ni Zhang^2,#^, Guohai Huang^3^, Weijin Zhang^4^, Qingnan Li^1^, Xuecong Ren^4,*^

^1^ Clinical Research Center, Shantou Central Hospital, Shantou, China.

^2^Metabolic Medicine Center, International Institutes of Medicine, the Fourth Affiliated Hospital, Zhejiang University School of Medicine, Yiwu, China.

^3^ Department of Blood Purification Center, Shantou Central Hospital, Shantou, China.

^4^ Department of Rheumatology and Immunology, Shantou Central Hospital, Shantou, China

^5^ Department of Geriatrics, Zhejiang Key Laboratory of Traditional Chinese Medicine for the Prevention and Treatment of Senile Chronic Diseases, Affiliated Hangzhou First People’s Hospital, School of Medicine, Westlake University, Hangzhou, China.

^6^ State Key Laboratory of Quality Research in Chinese Medicine, Macau University of Science and Technology, Macao, China.

^#^ These authors contributed equally to this study.

* Corresponding authors:

Xuecong Ren: [rxc880715@163.com](mailto:rxc880715@163.com);

Guoxin Huang: [hgvxin@163.com](mailto:hgvxin@163.com)

Table 1

The sequencing of the primers used in this study

| *Lactobacillus* | F: AGCAGTAGGGAATCTTCCA |
| --- | --- |
|  | R: CACCGCTACACATGGAG |
| *Bifidobacterium* | F: GCGTGCTTAACACATGCAAGTC |
|  | R: CACCCGTTTCCAGGAGCTATT |
| β-actin | F: TGTTACCAACTGGGACGACA |
|  | R: CTGGGTCATCTTTTCACGGT |
| dsrA | F: CTGCGAATATGCCTGCTACA |
|  | R: TGGTCGARCTTGATGTCGTC |
| iNOS | F: GTTCTCAGCCCAACAATACAAGA |
|  | R: GTGGACGGGTCGATGTCAC |
| Arginase 1 | F: TGGCTTGCGAGACGTAGAC |
|  | R: GCTCAGGTGAATCGGCCTTTT |
| YM 1 | F: TTATCCTGAGTGACCCTTCTAAG |
|  | R: TCATTACCCTGATAGGCATAGG |
| IL-4 | F: GGTCTCAACCCCCAGCTAGT |
|  | R: GCCGATGATCTCTCTCAAGTGAT |
| IL-10 | F: GCTCTTACTGACTGGCATGAG |
|  | R: CGCAGCTCTAGGAGCATGTG |
| IL-12 | F: ACTCTGCGCCAGAAACCTC |
|  | R: CACCCTGTTGATGGTCACGAC |
| TNF-α | F: CAAATGGCCTCCCTCTCAT |
|  | R: CTCCTCCACTTGGTGGTTTG |
| CXCL 10 | F: CCAAGTGCTGCCGTCATTTTC |
|  | R: GGCTCGCAGGGATGATTTCAA |
| IL-1β | F: GCTGAAAGCTCTCCACCTCA |
|  | R: GGCCACAGGTATTTTGTCGT |
| *A. muciniphila* | F: CAGCACGTGAAGGTGGGGAC |
|  | R: CCTTGCGGTTGGCTTCAGAT |
| Universal bacteria | F: ACTCCTACGGGAGGCAGCAGT |
|  | R: ATTACCGCGGCTGCTGGC |
| *D. vulgaris* | F: GGCATCTGTAGACCTCCTTGTAGTC |
|  | R: TGTCGATCGTAGGTAGCAAATGGCG |
